# Publisher Correction: A repeated cross-sectional and longitudinal study of mental health and wellbeing during COVID-19 lockdowns in Victoria, Australia

**DOI:** 10.1186/s12889-022-14963-3

**Published:** 2023-01-13

**Authors:** Annemarie Wright, Alysha De Livera, Keun Hee Lee, Carl Higgs, Matthew Nicholson, Lisa Gibbs, Anthony Jorm

**Affiliations:** 1 Department of Health, Victorian State Government, Melbourne, Australia; 2 Melbourne School of Population and Global Health, The University of Melbourne, Melbourne, Australia; 3 Mathematical Sciences, School of Science, RMIT University, Melbourne, Australia; 4 Department of Mathematics and Statistics, La Trobe University, Melbourne, Australia; 5 College of Design and Social Context, RMIT University, Melbourne, Australia; 6 Monash University Malaysia, Kuala Lumpur, Malaysia

**Correction: BMC Public Health 22, 2434 (2022)**

**https://doi.org/10.1186/s12889-022-14836-9**

During the publication [1] process an erroneous contribution statement was not correctly removed. This statement has now been corrected. The publisher apologizes for the inconvenience caused to the authors & readers.
